# Increasing Incidence, but Lack of Seasonality, of Elevated TSH Levels, on Newborn Screening, in the North of England

**DOI:** 10.4061/2010/101948

**Published:** 2010-01-28

**Authors:** Mark S. Pearce, Murthy Korada, Julie Day, Steve Turner, David Allison, Mohammed Kibirige, Tim D. Cheetham

**Affiliations:** ^1^Institute of Health and Society, Newcastle University, Newcastle upon Tyne NE1 4LP, UK; ^2^Department of Paediatrics, Royal Victoria Infirmary, Newcastle upon Tyne NE1 4LP, UK; ^3^Department of Biochemistry, University Hospital of North Durham, Durham DH1 5TW, UK; ^4^Department of Clinical Biochemistry, Royal Victoria Infirmary, Newcastle upon Tyne NE1 4LP, UK; ^5^Department of Microbiology, University Hospital of North Durham, Durham DH1 5TW, UK; ^6^Department of Paediatrics, James Cook University Hospital, Middlesbrough TS3 3TA, UK

## Abstract

Previous studies of congenital hypothyroidism have suggested an increasing incidence and seasonal variation in incidence, which may suggest nongenetic factors involved in aetiology. This study describes the incidence of elevated thyroid stimulating hormone (TSH) values in newborns, a surrogate for congenital hypothyroidism, measured as part of the screening programme for congenital hypothyroidism, over an eleven-year period (1994–2005), and assesses whether seasonal variation exists. All infants born in the Northern Region of England are screened by measuring levels of circulating TSH using a blood spot assay. Data on all 213 cases born from 1994 to 2005 inclusive were available. Annual incidence increased significantly from 37 per 100,000 in 1994 to a peak of 92.8 per 100,000 in 2003. There was no evidence of seasonal variation in incidence. The reasons for the increasing incidence are unclear, but do not appear to involve increasing exposure to seasonally varying factors or changes in measurements methods.

## 1. Introduction

Congenital hypothyroidism (CHT) is the most common congenital endocrine disorder with a world-wide incidence around 1 in 3500 to 4000 live births. Reduced thyroid hormone production in babies with CHT has a major detrimental effect on central nervous system development and growth. Prompt treatment with thyroxine will prevent these problems arising in the majority of babies.

Most babies with CHT have thyroid gland agenesis or dysgenesis with a poorly formed or absent gland. A minority (~20%) have a normally sited gland but an underlying single gene defect preventing the normal process of thyroid hormone production within the gland (dyshormonogenesis). Although germline mutations in thyroid transcription factors 1 and 2 (TTF-1 and TTF-2) and PAX-8 (paired box transcription-8) have been identified as aetiological risk factors for dysgenesis or agenesis, they only explain a small percentage of cases (around 2%) [1]. It is important to highlight the fact that iodine deficiency is a well recognised and important cause of neonatal thyroid dysfunction in some parts of the world. 

An increasing incidence of CHT has been suggested from an analysis of data including that from New York State [2] and Mexico [3], although no similar increase was shown in a similar study of data from Quebec [4]. Previous studies in the United Kingdom have suggested that CHT is more prevalent in Asian sectors of the population and that prevalence has increased [5]. Studies also suggest that the prevalence of hypothyroidism in Scotland has increased [6], with a study from the same area of Scotland demonstrating an increased population prevalence of hypothyroidism in young people compared to previously published rates [7]. 

The incidence of CHT has also been suggested to vary seasonally in a number of studies in different geographical areas including the West Midlands of England [5], Finland [8], Japan [9–11], and Australia [9]. However, seasonality also has been not observed in a number of other studies, including those in the North West of England [12], the Netherlands [13], Saudi Arabia [14], Canada [4], Norway, France and Switzerland [9]. 

Should seasonal variation in CHT risk exist, this would suggest that an unknown environmental factor may be involved in the disease's aetiology. Temporal trends in risk are usually unlikely to include genetic factors, unless either population shifts result in germline mutations being more prominent in a particular geographical area or environmental influences on germline mutations or epigenetic changes have increased in prevalence over the study period.

Circulating thyroid stimulating hormone (TSH) levels are measured as part of screening programmes for CHT across many parts of Europe, Japan, and increasingly in North America. In the UK, neonatal screening for CHT began in 1979 in Scotland and in 1981 in the remainder of the UK after a recommendation from the UK Department of Health [15].

This paper describes the incidence of elevated TSH levels in newborns in the North of England over an eleven-year period (1994–2005) and examines whether seasonal variation in incidence exists in this geographical area.

## 2. Methods

Around 35 000 infants in the Northern Region of England, comprising North East England (the area from Teesside extending north into Northumberland) and North Cumbria, are screened every year in a single centre by measuring blood spot TSH levels. Data on all cases, including dates of birth, were available from 1994 to 2005 inclusive.

### 2.1. TSH as a Surrogate for CHT

We opted to refer to “TSH” rather than “CHT” in this study for the following reasons.

1. The extent to which cases of suspected CHT are investigated will vary from one unit to another [16, 17]. We felt that this was likely to be the case in our region of the UK as well.
2. There is no definitive test or tests that can identify the underlying thyroid gland abnormality in this condition. Even the combination of isotope scanning and ultrasonography does not reveal an underlying diagnosis in all infants [16, 18].
3. The sensitivity and specificity of tests such as isotope scanning is suboptimal with potentially misleading information generated by factors such as early thyroxine therapy [18].
4. Some studies have used thyroxine intervention as confirmation of underlying CHT but the threshold for intervention will vary with time and from clinician to clinician and centre to centre [19]. Hence some babies with “raised” TSH values and thyroid hormone values within the laboratory normal range will be treated whilst others will not [5, 16].
5. Ultimately, biochemistry is the most important parameter; a baby with a raised TSH but normal imaging will require thyroxine treatment whilst a baby with normal biochemistry but abnormal imaging will not.

### 2.2. Sample Processing and Analysis

During the period of the study the screening centre moved between the Royal Victoria Infirmary (RVI), Newcastle and the University Hospital of North Durham (UHND), Durham. The screening blood spot TSH method and cut-off value for screening failure also changed during the study from a manual radioimmunassay method (1994–March 1998) to an ACS-(Beyer) chemiluminometric assay (April 1998–February 2003) and then a DELFIA (perkin Elmer) fluoroimmunometric assay (March 2003–present) (Table 1).

Interassay coefficients of variation (CV) for blood spot TSH assays were 16.5%, 9.4%, and 8.8% for the manual radioimmun assay method at TSH values of 22.0, 38.6, and 74.1 mU/L, respectively. Interassay CV for blood spot TSH assays were 7% and 6%  for the ACS chemiluminometric assay at TSH values of 14 mU/L and 68 mU/L, respectively, and 11% and 12% for the DELFIA fluoroimmunometric assay at TSH values of 16 mU/L and 60 mU/L, respectively. Nine replicates over 9 analytical runs were used to calculate inter assay precision for the ACS assay and 42 replicates over 10 analytical runs for the DELFIA assay. To ensure that cut-off values were comparable across the different screening methods used, prior to a change in method blood spot samples received as part of the screening program were analysed by both methods (Radioimmunoassay *v* ACS-180, *n* = 2634; ACS-180 *v* DELFIA, *n* = 682). Revised cut-off values were established by comparing the results using scatter charts and using least squares linear regression. There was a highly significant correlation between the 2 methods when the assay was changed from RIA to ACS-180 in 1998 (*P* < .001; *r* ^2^ = 0.94) and when it was changed from ACS-180 to DELFIA in 2003 (*P* < .001; *r* ^2^ = 0.99). 100% agreement for screening passes and failures was obtained using the revised cut-off values. 

Values identified as being greater than 20 mU/L by the radioimmunassay method, 10 mU/L by the ACS method, and greater than 6 mU/L by the DELFIA method were followed by analysis of a repeat blood spot from the screening card. They were deemed to be screening failures if the final blood spot value was again greater than 10 mU/L (ACS) or 6 mU/L (DELFIA). All infants where the paediatrician was subsequently notified of an increased value and hence were classed as neonatal screening test failures were included in the analysis.

### 2.3. Statistical Analysis

The yearly incidence of elevated TSH values in newborns was calculated as the number of cases in each year per 100,000 live births born in the Northern Region. Temporal changes in incidence were assessed using Poisson regression. Seasonal variation in incidence was assessed using the Edwards test for seasonality [20] with an adjustment for variable month length. A *P*-value of less than .05 was considered statistically significant. All statistical analyses were performed using the statistical software package Stata, version 9.0 (StataCorp, Texas).

Approvals for this study were obtained from the Newcastle and North Tyneside Local Research Ethics Committee and the Patient Information Advisory Group for England and Wales.

## 3. Results

Between 1994 and 2005 inclusive, there were 213 cases of elevated TSH values in newborns in the Northern Region of England. The ratio of female to male cases was 1.3 : 1. Over the study period, the average annual incidence was 59.94 per 100,000 live births. The annual number of cases of TSH elevation in newborns in the Northern Region and annual incidence per 100,000 live births are shown in Table 2. Incidence increased significantly over the study period (*P* < .0001) from 37 per 100,000 in 1994 to a peak of 92.8 in 2003.

The number of cases by month of birth is reported in Table 3. Despite peak number of cases in May and in the August–October period, there was no significant evidence of seasonal variation in the number of cases (*P* = .16). Nor was there evidence of seasonal variation with sex-specific seasonality analyses (*P* = .17 for females and 0.59 for males).

## 4. Discussion

Despite advances made in identifying genetic risk markers for CHT, there remains a great deal to be explained in terms of the aetiology of the disease [21]. This study showed an increasing incidence of elevated TSH values in newborns in the Northern Region of England between 1994 and 2005, but did not find evidence of seasonal variation in the number of cases. Many other studies have depended primarily on biochemistry including TSH rather than other investigations when making a diagnosis of CHT [22]. Given the high risk of subclinical hypothyroidism and morphological abnormalities in “false-positive” patients [23, 24] we suspect that our figures for raised TSH will be closely linked to the number of actual cases of CHT. Unfortunately we do not have detailed information on outcome in these children because they were managed in more than 10 different hospitals by an even greater number of clinicians. More detailed data were therefore not available to allow us to assess changes in permanent CHT or to analyse the data with respect to different aetiologies.

An increasing temporal trend in incidence of CHT has recently been reported in New York State [2], with a 138% increase between 1978 and 2005. Excluding New York State, nationwide United States data suggest a 73% increase between 1987 and 2002 [22]. We observed a 151% increase in raised TSH values between 1994 and 2003. However, this is in contrast to research conducted in Quebec, Canada, where no changes in incidence were seen over a 16-year period [4]. A real temporal trend, aside from changes in diagnostic procedures which can lead to increases in incidence [25], suggests either an increasing exposure to an environmental risk factor, or a changing distribution of risk factors among the population. The incidence rates in this study dropped slightly after 2003 and it remains to be seen whether this is a true decline or simply random variation. 

The division between a “screen positive” and “screen negative” in a screening programme such as this is not linked to robust outcome measures and the screening threshold and management of cases with mild thyroid dysfunction varies between regions. We were keen to establish that the change in incidence was not simply a reflection of change in assay methodology or laboratory practice (as opposed to seasonality where we would not expect assay change to have the same impact). All births in the study region were screened in a single centre at any one time. The physical location of this centre changed originally in April 1998 from Newcastle to Durham and again in April 2005 from Durham to Newcastle. The move in 1998 also corresponded to a change in the laboratory assay with a further change in assay in 2003. The different assay methods were rigorously compared to ensure that there would be no difference in the number of cases identified as a result of the change. To this end a large number of samples were analysed and there was no difference in screening passes or failures with 100% concordance. It is of note that the increasing incidence in raised TSH values was most striking during the period when TSH was measured only by the ACS method although we suspect that this represents a true increase because the rise had commenced prior to the change in methodology and continued after this changed to the DELFIA assay. Many other studies of CHT or elevated TSH levels have encountered similar issues regarding data interpretation as assay methodology has changed [16].

In terms of changes in the population structure, two previous studies from England have reported an increased incidence of CHT among Asians [5, 12]. However, the Northern Region of England has a population of 3.1 million, of which less than 2% are from ethnic minorities, with low levels of migration [26]. Therefore, while the data are not available to assess directly, it is unlikely that the increased incidence is related to changes in population structure and with it changes in genetic risk factor profiles. Exposure to environmental factors such as chemicals or increasing levels of other risk factors such as maternal iodine deficiency or high prenatal iodine exposures [27] or low birth weight infants [28] may be suggested by an increasing temporal trend whereas infections or seasonally varying dietary factors or chemical exposures, such as dioxin and polychlorinated biphenyl [29], may be suggested by evidence of seasonal variation in the number of cases. The potential role of a suboptimal maternal iodine status in some parts of the North of England should be highlighted and clearly warrants further study [30]. We found little evidence of seasonal variation of elevated TSH levels in newborns, in line with a number of previous reports of no seasonality [4, 9, 12–14]. In contrast, a number of previous studies have reported seasonal variation in a number of different geographical areas [5, 8–11], including a different part of England [5]. Gu et al. also found sex-specific seasonal patterns of incidence in Japan [31]. However, sex-specific analyses also showed little evidence of seasonal variation in this study. The issue of statistical power should be considered when interpreting our results and it is possible that with a larger sample a seasonal effect may have been found. It is also possible that differences in findings may reflect differences in the underlying populations. Our sex ratio (F : M) of 1.3 : 1 was significantly less than the sex ratio of 2.8 : 1 previously shown in a study from Scotland that used thyroxine prescription data as a surrogate for hypothyroidism in children and young people [7] and less than the ratio of 2.1 : 1 reported for cases of “true” CHT from the same country [16]. This underlines the importance of taking factors such as iodine status into consideration in future work.

In conclusion, we have observed a significant increasing trend in the incidence of elevated TSH levels in newborns, a surrogate for increasing levels of CHT, since 1994. Whilst the reasons for the increase are unclear, it would appear from this analysis that seasonally varying factors are not involved. It is also unlikely to be due to a change in the population distribution of genetic risk factors, although environmental determinants of genetic mutations and epigenetic factors cannot be ruled out. Further research is required into the potential environmental determinants of increased CHT risk.

## Figures and Tables

**Table 1:** TSH newborn screening base, assay methodology, and cut-off values during the study period.

	1994–March 1998	April 1998–February 2003	March 2003–March 2005	April 2005–present
Centre	Newcastle	Durham	Durham	Newcastle
Method	Radioimmunoassay	ACS-180	DELFIA	DELFIA
	(Manual)	(Bayer)	(Perkin Elmer)	(Perkin Elmer)
TSH cut-off (mU/L)	20	10	6	6

**Table 2:** Annual number of cases and incidence of elevated TSH levels on newborn screening in the Northern Region of England, 1994–2005.

Year	Number of cases	Incidence per 100,000 live births
1994	15	37.12
1995	11	32.26
1996	13	38.61
1997	5	15.21
1998	16	49.99
1999	20	64.65
2000	15	50.63
2001	22	76.11
2002	26	89.02
2003	28	92.84
2004	22	70.93
2005	20	63.64

**Table 3:** Number of cases of elevated TSH levels on newborn screening by month of birth in the Northern Region of England, 1994–2005.

Month	Number of cases
January	14
February	14
March	14
April	17
May	25
June	19
July	16
August	20
September	23
October	20
November	16
December	15
